# De novo assembly and characterization of the first draft genome of quince (*Cydonia oblonga* Mill.)

**DOI:** 10.1038/s41598-021-83113-3

**Published:** 2021-02-15

**Authors:** Aysenur Soyturk, Fatima Sen, Ali Tevfik Uncu, Ibrahim Celik, Ayse Ozgur Uncu

**Affiliations:** 1 Department of Molecular Biology and Genetics, Necmettin Erbakan University, Meram, Konya 42090 Turkey; 2 Department of Biotechnology, Necmettin Erbakan University, Meram, Konya 42090 Turkey; 3 Department of Agricultural and Livestock Production, Pamukkale University, Denizli, 20700 Turkey

**Keywords:** Agricultural genetics, Genomics

## Abstract

Quince (*Cydonia oblonga* Mill.) is the sole member of the genus *Cydonia* in the Rosacea family and closely related to the major pome fruits, apple (*Malus domestica* Borkh.) and pear (*Pyrus communis* L.). In the present work, whole genome shotgun paired-end sequencing was employed in order to assemble the first draft genome of quince. A genome assembly that spans 488.4 Mb of sequence corresponding to 71.2% of the estimated genome size (686 Mb) was produced in the study. Gene predictions via ab initio and homology-based sequence annotation strategies resulted in the identification of 25,428 and 30,684 unique putative protein coding genes, respectively. 97.4 and 95.6% of putative homologs of Arabidopsis and rice transcription factors were identified in the ab initio predicted genic sequences. Different machine learning algorithms were tested for classifying pre-miRNA (precursor microRNA) coding sequences, identifying Support Vector Machine (SVM) as the best performing classifier. SVM classification predicted 600 putative pre-miRNA coding loci. Repetitive DNA content of the assembly was also characterized. The first draft assembly of the quince genome produced in this work would constitute a foundation for functional genomic research in quince toward dissecting the genetic basis of important traits and performing genomics-assisted breeding.

## Introduction

*Cydonia* is a monospecific genus in the Rosaceae family with quince (*Cydonia oblonga* Mill.) (2n = 2x = 34) identified as the sole species within the genus^1,2^. Quince is a deciduous small tree, usually growing to a height of less than 5 m^3^. *Cydonia* genus was named after the Kydonia region at the northwestern coast of Crete, Greece, where quince has long been cultivated^4^. Caucasus region is the probable center of origin for quince and domestication of the plant dates back to 5000 BC. Quince was introduced to Greece and the Roman Empire around 600 and 200 BC, respectively. The tree arrived at the Americas from Europe in seventeenth century AD^2^. *C. oblonga* is classified in the apple tribe (Maleae), which includes several widely cultivated members such as apples (*Malus* Mill.), pears (*Pyrus* L.), serviceberries (*Amelanchier* Medik.), chokeberries (*Aronia* Medik.) and loquats (*Eriobotrya* Lindl.)^5,6^. Maleae members share the common basic chromosome number of x = 17 except the *Vauquelinia* genus (x = 15)^6^.

Most of the quince fruit production is for the food industry as the fruits are used for producing marmalades, jams, sweets, liqueurs and aromatic distillates^7^. Quince is also recognized as a cheap and rich source of health-beneficial secondary metabolites. Leaf infusions/decoctions have long been used as a herbal remedy and the fruit is a proposed source for extracting health-promoting phytochemicals for pharmaceutical and nutraceutical applications^7^. Besides cultivation for fruit production, the primary importance of quince tree is its utilization as a dwarfing rootstock for pear cultivation^3^. Quince rootstocks limit tree size for easy harvest, and improve productivity and fruit quality^2,4,8^. For example, ‘Quince A’ is a well-known, commercially propagated rootstock for pear production^3,8^.

There is a limited number of molecular genetic studies conducted on quince. Accordingly, a search that covers all NCBI databases returns only 272 and 129 entries labeled as ‘*Cydonia oblonga*’ in ‘Nucleotide’ and ‘Gene’ databases, respectively (https://www.ncbi.nlm.nih.gov/search/all/?term=cydonia%20oblonga; Access date: December 2020). In contrast, genome sequences are available for apple and pear, the two related pome fruit species^9–11^. Due to the lack of quince genomic resources, molecular genetic studies on quince are mostly dedicated on germplasm characterization with transferable molecular genetic tools, namely SSR (simple sequence repeat) markers developed for apple and/or pear genomes^8,12–14^. In other work, generic, random marker systems including ISSR (inter simple sequence repeat)^4^, RAPD (random amplified polymorphic DNA)^15^ and AFLP (amplified fragment length polymorphism)^16^ were utilized for the molecular genetic characterization of quince collections. Genome assemblies provide the foundation for further extensive molecular genetic research in agriculturally relevant species. In the present work, a draft genome assembly of quince was produced for the first time using whole genome shotgun paired-end sequencing. Assembly characterization was performed by ab initio and homology-based gene predictions, as well as microRNA coding loci identification employing machine-learned classification. Repetitive portion of the assembly was characterized by the analyses of transposable element content and microsatellite composition.

## Materials and methods

### DNA isolation, sequencing and sequence pre-processing

DNA was extracted from leaf tissue of *C. oblonga* rootstock clone ‘Quince A’ using a modified CTAB protocol^17^ as follows: 200 mg liquid nitrogen frozen, ground leaf tissue was mixed with 800 μL of CTAB extraction buffer [100 mM Tris–HCl (pH 8.0), 20 mM EDTA (pH 8.0), 1.4 M NaCl, 2% (w/v) CTAB, 1% PVP] and 5 μL of RNase A (0.01 mg/μL), and incubated at 65 °C for 1 h. Lysed sample was mixed with 600 μL of chloroform:isoamyl alcohol (24:1) and centrifuged at 20,900×*g* for 10 min. The supernatant phase was incubated with 200 μL of isopropanol at 4 °C for 1 h for DNA precipitation. DNA pellet was collected by centrifugation at 4 °C for 10 min at 20,900×*g*, washed with 100 μL of 70% ethanol and re-suspended in 100 μL of Tris–EDTA buffer (pH 8.0).

Paired-end sequencing was provided by Macrogen NGS Service (Macrogen Inc., Korea) using an Illumina NovaSeq 6000 platform. A paired-end sequencing library of median insert size of 450 bp was prepared using a TruSeq DNA PCR-Free kit according to TruSeq DNA PCR-Free Sample Preparation Guide prior to sequencing. Reads of 151 bp length were obtained as a result of paired-end sequencing. Data were filtered for reads that pass the Q30 score (92.77% of the total reads) and barcode adapters were trimmed using FASTX-Toolkit version 0.0.13 (http://hannonlab.cshl.edu/fastx_toolkit/index.html).

### Sequence assembly and quality evaluation

Short reads were assembled using SOAPdenovo2 version 2.04^18^ with a k-mer length of 127 and filtered for a minimum read length of 500 bp. Assembly completeness based on gene content was assessed using the BUSCO (Benchmarking Universal Single-Copy Orthologs) pipeline^19^. The lineage dataset used was eudicots_odb10 (Creation date: 2019-11-20, number of species: 31, number of BUSCOs: 2326). Contigs that represent the plastid genome were aligned to the *C. oblonga* plastid genome assembly (GenBank Accession: NC_045415.1).

### Gene prediction and functional annotation

Gene prediction was performed using both ab initio and homology-based strategies. Ab initio gene prediction was performed using the AUGUSTUS gene prediction server^20^ via the OmicsBox version 1.3 platform (https://www.biobam.com/omicsbox/). Assembled *C. oblonga* genome sequences were used as the input for ab initio annotation process. Repeat Masking application via RepeatMasker Open-4.0^21^ under the Genome Analysis module of OmicsBox was run prior to gene prediction. Functional annotation was performed using the output file of the ab initio gene prediction process. Accordingly, the UniProt collection of apple (*Malus domestica* Borkh.) protein sequences (45,359 sequences) (https://www.uniprot.org/uniprot/?query=malus+domestica&sort=score; Access date: September 2020) and plant transcription factor database (PlnTFDB version 3.0)^22^ were used to create local databases for running blastp searches. The E-value threshold used was 1E−10. GO mapping was performed on the output data and GO terms were assigned to the predicted genes with the subsequent GO annotation process. Homology-based gene prediction was also performed with an E-value threshold of 1E−10 based on *M. domestica* proteins using the genome assembly file as the input and the UniProt collection of *M. domestica* protein sequences as the local database.

### miRNA coding loci identification

Ab initio pre-miRNA (precursor microRNA) detection employing a machine learning algorithm and homology-based pre-miRNA search were used in combination to predict pre-miRNA coding loci in *C. oblonga* genome assembly. Toward this aim, 7579 plant pre-miRNA sequences available on miRbase (Release 22.1)^23^ were downloaded and used for training and testing classifiers. Alongside the positive pre-miRNA dataset, a negative training and testing sample set was used that consisted of plant protein coding sequences. Pre-miRNAs and the negative sequence dataset were parameterized using k-mer (k = 4) method^24^. Frequency of each k-mer in the input sequence was detected and used to create a matrix as the parameterized version of the biological sequences. k-mers with unambiguous base calls (N, R, Y, S, W, etc.) were filtered out from the data. Datasets were split into training (70%) and testing data (30%) for training classifiers and evaluating their performance. Random forest (RF), Support Vector Machine (SVM), Naïve Bayes (NB), XGBoost, K-Nearest Neighbors (KNN) classifiers were trained and tested for their performance using the metrics of precision, accuracy, recall (sensitivity) and F-measure according to the following Eqs. (1–4) (TP, true positive; FP, false positive; TN, true negative; FN, false negative)^25^:

1 $$\text{Precision}=\frac{\text{TP}}{\text{TP}+\text{FP}}$$

2 $$\text{Accuracy}=\frac{\text{TP}+\text{TN}}{\text{TP}+\text{TN}+\text{FN}+\text{FP}}$$

3 $$\text{Recall}=\frac{\text{TP}}{\text{TP}+\text{FN}}$$

4 $$\text{F} \;\text{measure}=2*\frac{\text{precision}*\text{recall}}{\text{precision}+\text{recall}}$$

SVM was the best performing machine-learned model for pre-miRNA prediction and used for the de novo identification of putative miRNA coding loci in the assembly. The trained SVM classifier was run on Scikit-learn 0.23.2 library^26^ in order to scan the assembly with a predefined sliding window of 70 nucleotides. The output of SVM classification was used for the homology search with plant pre-miRNA sequences deposited in miRBase (7579 sequences). The E-value threshold used for the homology search was 1E−10 with a word size of 7.

### Characterization of transposable elements (TEs) and microsatellite composition

Domain Based Annotation of Transposable Elements (DANTE) was performed via the RepeatExplorer server^27^ (Access date: September 2020) for the identification and characterization of the transposable element content by domain search and subsequent phylogenetic annotation and classification. Microsatellite composition was analyzed using GMATA version 2.01 (Genome-wide Microsatellite Analyzing Tool Package)^28^. Mononucleotide repeat loci of minimum 10 bases and 2–6 nucleotide microsatellite motifs of min. 5 repeats were mined in the assembly.

## Results and discussion

### Genome assembly and quality evaluation

Paired-end sequencing produced 494.5 million reads of 151 bp length. The total size of the raw sequence reads was 74.7 Gb. Sequence Read Archive (SRA) files are deposited at GenBank under the BioProject PRJNA675337. Relative GC and AT content of the sequence reads was 38.7 and 61.3%, respectively. Ratio of bases with phred quality score over 20 (Q20) was 97.1% and the ratio with quality score over 30 (Q30) was 92.8% (Table 1). Assembly of the barcode-trimmed reads that pass the Q30 filter produced 303,932 contigs larger than 500 bp. The total size of the assembly was 488.4 Mb, corresponding to 71.2% of the *C. oblonga* genome size (686 Mb) estimated by flow cytometry^29^. N50 value of the assembly was calculated as 2.4 kb and the maximum contig size was 53.8 kb (Table 1). Assembly files are deposited at GenBank under the accession JADOBS000000000.

**Table 1:** Sequencing and assembly statistics.

Raw data and assembly statistics	
Number of reads	494,494,378
Number of read bases (bp)	74,668,651,078
GC content (%)	38.66
AT content (%)	61.34
Q20 (%)	97.14
Q30 (%)	92.77
Read coverage	108x
Number of contigs	303,932
Min. contig length (bp)	500
Max. contig length (bp)	53,821
N50 value (bp)	2435
Total assembly length (bp)	488,422,409
Total number of BUSCOs	1312
Complete BUSCOs	1024
Fragmented BUSCOs	288

Searching BUSCO sets enables the quantitative assessment of genome completeness using an evolutionary measure, informed expectation of orthologous gene content^19^. Assessing the assembly completeness via BUSCO identified 1312 BUSCOs (81.2%) out of 1614 BUSCO groups of the Embryophyta database. 1024 of the identified BUSCOs were complete BUSCOs, 869 of which were complete single-copy and 155 were complete duplicated orthologs. 288 out of 1312 BUSCOs were fragmented. The number of missing BUSCOs in the genome assembly was 302 (18.8%). Complete list of BUSCO IDs, identification status of each ID, gene locations in the assembly and gene descriptions are provided as Supplementary Table S1.

### Functional annotation of protein coding gene content

**Figure 1:**
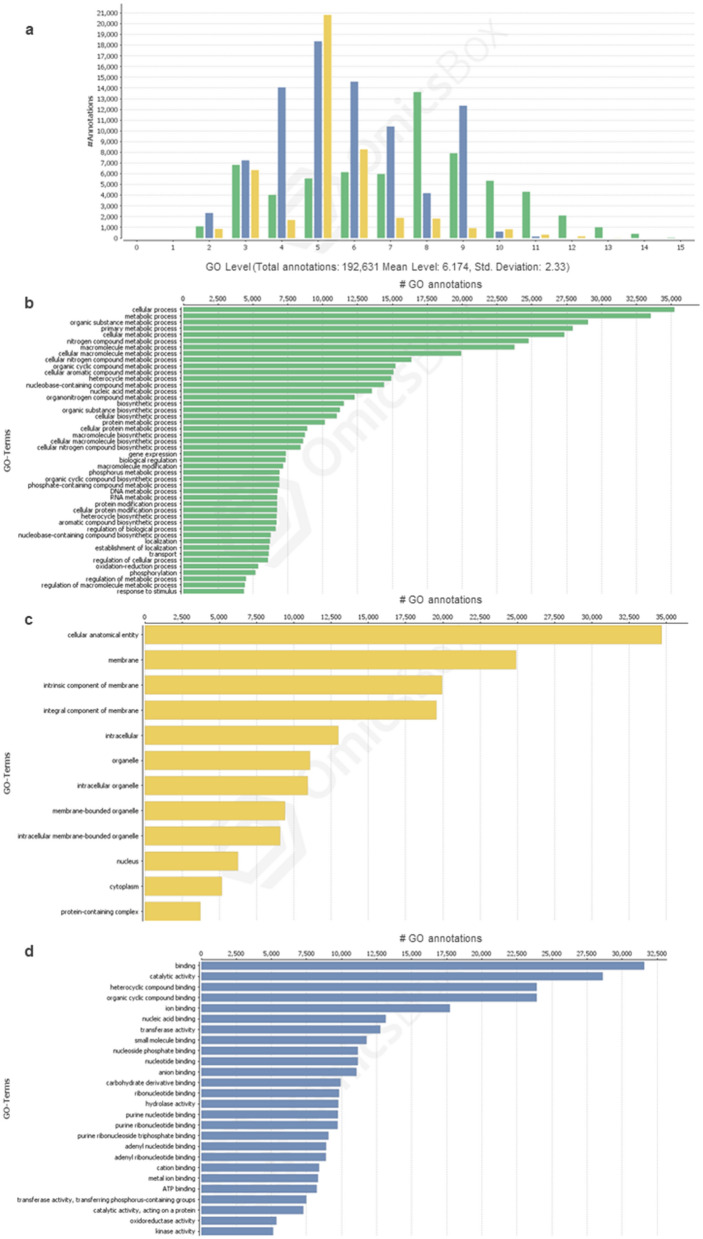
Distribution of GO annotation levels and sequence GO term assignments. (**a**) GO level distribution of the annotations assigned to the three categories, biological process (BP), cellular component (CC) and molecular function (MF). Green, yellow and blue represent BP, CC and MF categories, respectively. (**b**) Distribution of annotations according to GO terms assigned to the BP category. (**c**) Distribution of annotations according to GO terms assigned to the CC category. (**d**) Distribution of annotations according to GO terms assigned to the MF category.

Ab initio gene prediction based on a generalized hidden Markov model combining signal and content sensors^20^ produced gene sequence predictions. Gene features file describing genes and gene features in assembly contigs can be accessed at 10.6084/m9.figshare.13538942. Predicted genic sequences were further matched with annotated *M. domestica* proteins, corresponding to 25,428 unique UniProt IDs (Supplementary Table S2). Direct homology-based annotation was also performed in addition to ab initio gene prediction, and identified 30,684 putative genes based on homology with *M. domestica* proteins (Supplementary Table S3). As a result of GO (gene ontology) mapping and annotation of ab initio predicted genic sequences, a total of 192,631 GO annotations were obtained for the three categories, biological process (BP), cellular component (CC) and molecular function (MF) at a mean level of 6.2 (Fig. 1a). The highest number of GO term assignments was obtained for ‘cellular process’ in the BP category (Fig. 1b). ‘cellular and anatomical entity’ was the top GO term for the CC category and ‘binding’ was the top molecular function according to the number of identified GO terms in the MF category (Fig. 1c,d). Sub categorization of the top GO terms at level 3 is provided as Supplementary Fig. S1.

**Figure 2:**
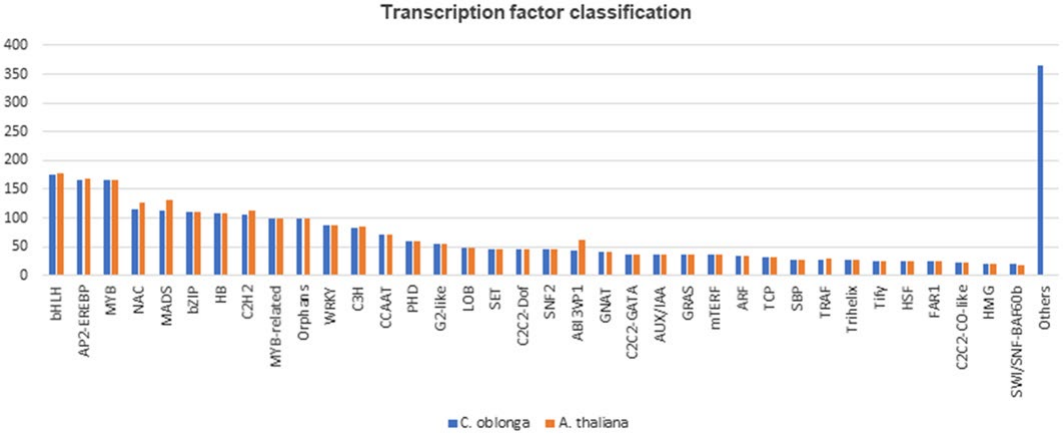
Distribution of *C. oblonga* transcription factors (TFs) based on plant TF families. *C. oblonga* TF distribution is displayed with respect to the distribution of Arabidopsis homologs. x-axis displays TF families and y-axis displays number of TF assignments to families. TF families with less than 20 representations in the set of identified *C. oblonga* TFs are included in the ‘Others’ class.

Transcription factors (TFs) have pivotal functions in almost all plant biological processes from seed germination to stress responses, therefore, play important roles in plant evolution and domestication^30^. A specific search for transcription factors in the ab initio predicted genic sequences was performed using the plant transcription factor database (PlnTFDB)^22^. The results were filtered for two species, *Arabidopsis thaliana* L. and *Oryza sativa* L. ssp. japonica. Arabidopsis and rice were specifically selected for defining the transcription factor composition in the assembly since they are the basic model species for eudicot and monocot genomics with well-annotated genomes and parallel expansion of TF orthologous groups in the two species has already been demonstrated^31^. PlnTFDB search identified 2686 of the 2757 annotated Arabidopsis TFs and 2981 out of 3119 annotated rice TFs in the assembly. Thus, 97.4 and 95.6% of the putative homologs of Arabidopsis and rice TFs were found to be present in the assembly. Putative homologs of the three largest TF families, bHLH (basic helix-loop-helix), MYB and AP2-EREBP (APETALA2-ethylene responsive element binding protein) were almost completely identified in the assembly with 175/177 bHLH, 166/166 MYB and 166/168 AP2-EREBP homologies (Fig. 2). The complete list of identified putative TF homologs is provided as Supplementary Table S4.

### Prediction of miRNA coding loci

**Figure 3:**
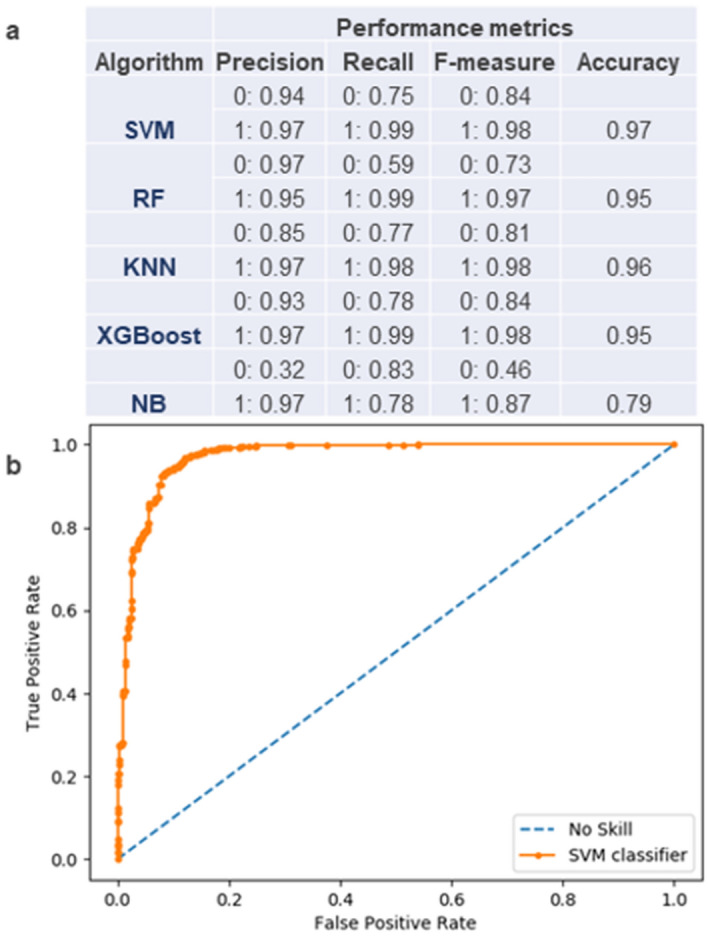
Performance evaluation of machine-learning algorithms for pre-miRNA identification. (**a**) Performance metrics are provided for the five tested algorithms: SVM, Support Vector Machine; RF, Random forest; KNN, K-Nearest Neighbors; XGBoost and NB, Naïve Bayes. Precision, Recall and F-measure values are calculated for classifying negative (0) and positive (1) test data to expected classes. (**b**) ROC (receiver operating characteristic) curve for the trained SVM classifier.

MicroRNAs regulate gene expression post-transcriptionally, playing crucial roles in plant development and stress responses. Therefore, during the last two decades, much effort has been devoted to miRNA identification^32^. The conventional miRNA identification route that relies on RNA sequencing can be biased toward abundant transcripts in addition to the dependence on tissue and developmental stage^33,34^. Thus, it is challenging to detect miRNAs that are constitutively expressed at low levels or expressed at specific tissues at very narrow time intervals^33^. Ab initio/de novo miRNA detection using genome scale sequences circumvents these problems and has proved successful in identifying pre-miRNAs in plant genomes^24,33,35,36^. As a result of related work, Support Vector Machine (SVM) has been reported as an effective machine-learned classifier to locate pre-miRNA coding loci in genome scale sequences^33,35,37^. In the present work, five different machine-learning algorithms (SVM, RF, KNN, XGBoost and NB) were trained and tested for their performance in predicting pre-miRNA coding genomic sequences. SVM was the best performing algorithm according to the applied performance metrics of accuracy, and F-measure (includes precision and recall measures) (Fig. 3a). The ROC (receiver operating characteristic) curve for the trained SVM classifier is provided as Fig. 3b. According to the results of the classifier performance test, the *C. oblonga* genome assembly was scanned with the trained SVM classifier, resulting in the identification of 600 putative pre-miRNA coding loci (Supplementary Table S5). The subsequent homology search using the SVM predicted loci as the query and the miRBase record of plant pre-miRNAs as the database, identified 33 matches including 28 pre-miRNAs from *M. domestica*, two from *Glycine max*, two from *Vitis vinifera* and 1 pre-miRNA from *Paeonia lactiflora* (Table 2).

**Table 2:** Results of miRNA prediction with the combined approach of SVM classification and homology-based identification.

Homologous miRNA^a^	Probability^b^	E-value^c^
mdm-MIR10978a	0.9954324	1.00E−20
mdm-MIR10979	0.99519524	6.00E−29
mdm-MIR10981a	0.99531038	1.00E−11
mdm-MIR10981b	0.99699497	9.00E−13
mdm-MIR10981c	0.99998466	1.00E−11
mdm-MIR10981d	0.9952244	4.00E−11
mdm-MIR10982c	0.99562967	1.00E−21
mdm-MIR10985	0.99575234	2.00E−23
mdm-MIR10990	0.99624388	2.00E−14
mdm-MIR10991e	0.99572793	9.00E−13
mdm-MIR10993a	0.99548501	2.00E−13
mdm-MIR10993b	0.99591172	1.00E−11
mdm-MIR10993f.	0.99683035	2.00E−13
mdm-MIR10995	0.99678045	2.00E−14
mdm-MIR11000	0.99545193	2.00E−13
mdm-MIR11002a	0.99541374	2.00E−19
mdm-MIR11006	0.99526081	1.00E−11
mdm-MIR11007	0.99557012	4.00E−11
mdm-MIR11008	0.99528467	4.00E−16
mdm-MIR11009	0.99734578	1.00E−15
mdm-MIR11014	0.99552524	1.00E−11
mdm-MIR11015	0.99547712	4.00E−11
mdm-MIR159d	0.9961144	1.00E−25
mdm-MIR164d	0.99998259	4.00E−11
mdm-MIR3627c	0.99623606	9.00E−13
mdm-MIR3627d	0.99520933	4.00E−16
mdm-MIR396f.	0.9951471	2.00E−13
mdm-MIR399b	0.99565726	1.00E−15
pla-MIR11602	0.99565912	4.00E−26
vvi-MIR171a	0.99652471	4.00E−11
vvi-MIR171g	0.99608369	5.00E−15
gma-MIR5032	0.99616243	4.00E−16
gma-MIR5368	0.99628882	5.00E−15

^a^pre-miRNA records from miRBase. mdm: *Malus domestica*; pla: *Paeonia lactiflora*; vvi: *Vitis vinifera*; gma: *Glycine max.*

^b^Trained SVM classifier probability value.

^c^E-value threshold applied for the BLAST analysis.

### Transposable element and microsatellite composition of the *C. oblonga* genome

**Figure 4:**
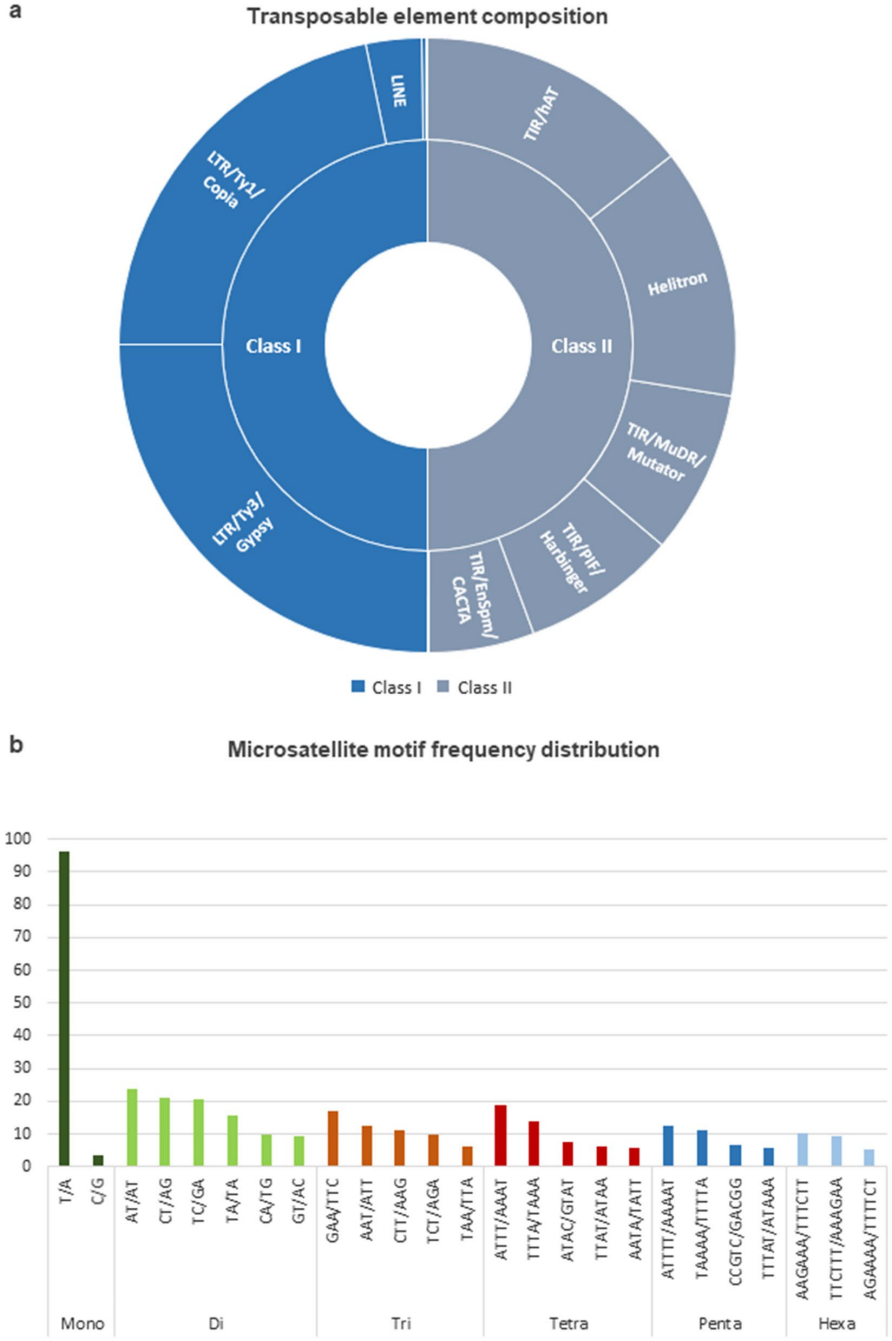
Repetitive DNA composition in the *C. oblonga* genome assembly. (**a**) Transposable element (TE) families are displayed in the doughnut chart according to their relative abundances in the assigned classes (Class I/II). Class I and Class II are not scaled to their relative occurrences in the *C. oblonga* genome, the abundances of TE families adding up to 100% for each class (Class I/II). TE families constituting less than 1% of either class is not displayed in the chart. (**b**) Microsatellite motif distribution is presented with respect to motif length. x-axis displays motifs that constitute more than 5% of the relevant motif type. y-axis displays percentage abundance of each motif.

TEs actively contribute to eukaryotic genome evolution^38^ and often modify peripheral gene expression via altered epigenetic marks. Such transposon induced gene expression patterns may directly be involved in the natural and/or artificial selection of certain advantageous genotypes in plant species. For example, red skin color in apple is subject to both natural and artificial selection and, a recent study associates the trait with a LTR (long terminal repeat) retrotransposon insertion upstream of the *MdMYB1* gene, which is a core transcriptional regulator of the anthocyanin biosynthesis pathway^11^. In the present work, the TE composition of the *C. oblonga* genome assembly was characterized using a domain-based approach which includes domain search followed by phylogenetic TE annotation and classification. As a result, 104,710 TEs were identified including 96,153 Class I and 8444 Class II elements (Table 3). Thus, the vast majority of TEs were retrotransposons, constituting 91.8% of the total number of TEs identified in the *C. oblonga* genome assembly. Most of the Class I elements were classified either as LTR/Ty3/Gypsy or LTR/Ty1/Copia elements (Fig. 4a) and constituted 93.6% of the total retrotransposon content. LTR superfamily transposons are the predominant TE type in many plant genomes^9–11,39–44^ and according to our TE composition analysis, quince genome is no exception with 85.9% of the total TE content identified as LTRs (Table 3). Class II elements constituted 8.1% of the total number of identified TEs. Helitrons and hAT superfamily transposons were the two most abundant DNA transposons, representing 26.3 and 29% of the Class II transposable elements. Out of the 104,710 TEs identified in the assembly, 113 loci (0.1%) were classified as ‘ambiguous’ since their phylogenetic assignment could not be inferred (Table 3). Detailed information on the identified TEs including the subfamily assignments is provided as Supplementary Table S6.

**Table 3:** TE composition in *C. oblonga* genome assembly.

	Abundance in the assembly	Frequency in total TEs	Frequency in defined class
**Class I**			
LTR/Ty3/Gypsy	48,178	46.01	50.11
LTR/Ty1/Copia	41,781	39.9	43.45
LINE	5594	5.34	5.82
Pararetrovirus	453	0.43	0.47
Unclassified	147	0.14	0.15
**Class II**			
Helitron	2224	2.12	26.34
TIR/hAT	2446	2.34	28.97
TIR/MuDR/Mutator	1452	1.39	17.2
TIR/PIF/Harbinger	1377	1.32	16.3
TIR/EnSpm/CACTA	931	0.89	11.03
TIR/Novosib	8	0.01	0.09
TIR/unclassified	6	0.01	0.07
Ambiguous	113	0.1

Quince genome assembly was also characterized for microsatellite composition. Search parameters were applied to identify mononucleotide repeats and tandem repeats of 2-to-6 nucleotides. As a result, a total of 308,171 microsatellite loci were identified in the assembly, corresponding to an overall density of 1.6 kb/microsatellite interval. Microsatellite motifs and positions in the assembly are provided as Supplementary Table S7. 171,414 of the identified loci were mononucleotide repeats and 136,757 loci were repeats of 2-to-6 nucleotide motifs (Table 4). Similar to the results of the microsatellite search in the quince genome, mononucleotide repeats appear as the most abundant repeat type in several plant genomes in case they are included in repeat mining analyses^45,46^. Stretches of A/T repeats predominated in the mononucleotide repeats, constituting 96.4% of the total number of identified mononucleotide microsatellites (Fig. 4b). Dinucleotide repeats (117,091 loci) were the most abundant microsatellite type in the pool of 2-to-6 nucleotide microsatellites (Table 4). The most frequent dinucleotide motif was AT (27,583 loci), representing 23.6% of the total number of dinucleotide microsatellites (Fig. 4b). Overall, AT-rich repeats predominated in *C. oblonga* genomic microsatellites (Fig. 4b). These results were consistent with work where overall microsatellite composition of the closely related apple genome was characterized^47^.

**Table 4:** Microsatellite abundance in *C. oblonga* genome assembly.

Motif length	Number of occurences	Frequency (%)
Mononucleotide	171,414	55.62
Dinucleotide	117,091	38
Trinucleotide	15,268	4.95
Tetranucleotide	3081	1
Pentanucleotide	833	0.27
Hexanucleotide	484	0.16
Total	308,171	100

## Conclusion

Quince is a neglected member of the Rosaceae family in terms of genomic studies. The species is closely related to the major pome fruits, apple (*M. domestica*) and pear (*P. communis*). Quince fruits are processed to different food products, liqueurs and aromatic distillates and, quince tree is a common rootstock for pear production. Yet, genomic research in quince has lagged far behind other pome fruit species. As a result of the present study, the first draft genome of quince was assembled from whole genome shotgun paired-end reads. Gene content was characterized by ab initio and homology-based gene predictions. Machine-learned classification methods were tested and applied for pre-miRNA coding loci predictions. Our results identified SVM as an appropriate machine-learning algorithm for de novo genomic pre-miRNA coding loci prediction. Transposable element content was characterized with a domain-based search and phylogenetic classification approach, identifying a very similar transposon composition with the closely related and well-characterized apple genome. Microsatellite content of the genome assembly was also analyzed and reported. Data produced in the present work provide insights into the quince genome for the first time and constitute a basis for further functional genomic research in quince.

## Supplementary Information

Supplementary Figure S1.

Supplementary Table S1.

Supplementary Table S2.

Supplementary Table S3.

Supplementary Table S4.

Supplementary Table S5.

Supplementary Table S6.

Supplementary Table S7.

## Data Availability

This Whole Genome Shotgun project has been deposited at DDBJ/ENA/GenBank under the accession JADOBS000000000, BioProject PRJNA675337. Sequence Read Archive (SRA) files have been deposited under the same BioProject (PRJNA675337). Gene features file describing the de novo predicted genes in gff format can be accessed at 10.6084/m9.figshare.13538942. Assembly characterization data generated during this study are included in this published article as Supplementary Information files.
