# Supplementary material for: De novo assembly and characterization of the first draft genome of quince (Cydonia oblonga Mill.)
Source: Sci Rep. 2021 Feb 15;11:3818. doi: 10.1038/s41598-021-83113-3 (PMC7884838; doi:10.1038/s41598-021-83113-3)

De novo assembly and characterization of the first draft genome of quince (*Cydonia oblonga* Mill.)

Aysenur Soyturk[1], Fatima Sen[2], Ali Tevfik Uncu[1], Ibrahim Celik[3], Ayse Ozgur Uncu[2,*]

[1]Department of Molecular Biology and Genetics, Necmettin Erbakan University, Meram, Konya, 42090, TURKEY

[2]Department of Biotechnology, Necmettin Erbakan University, Meram, Konya, 42090, TURKEY

[3]Department of Agricultural and Livestock Production, Pamukkale University, Denizli, 20700, TURKEY

Figure 1. Sub-categorization of the top GO terms at level 3.

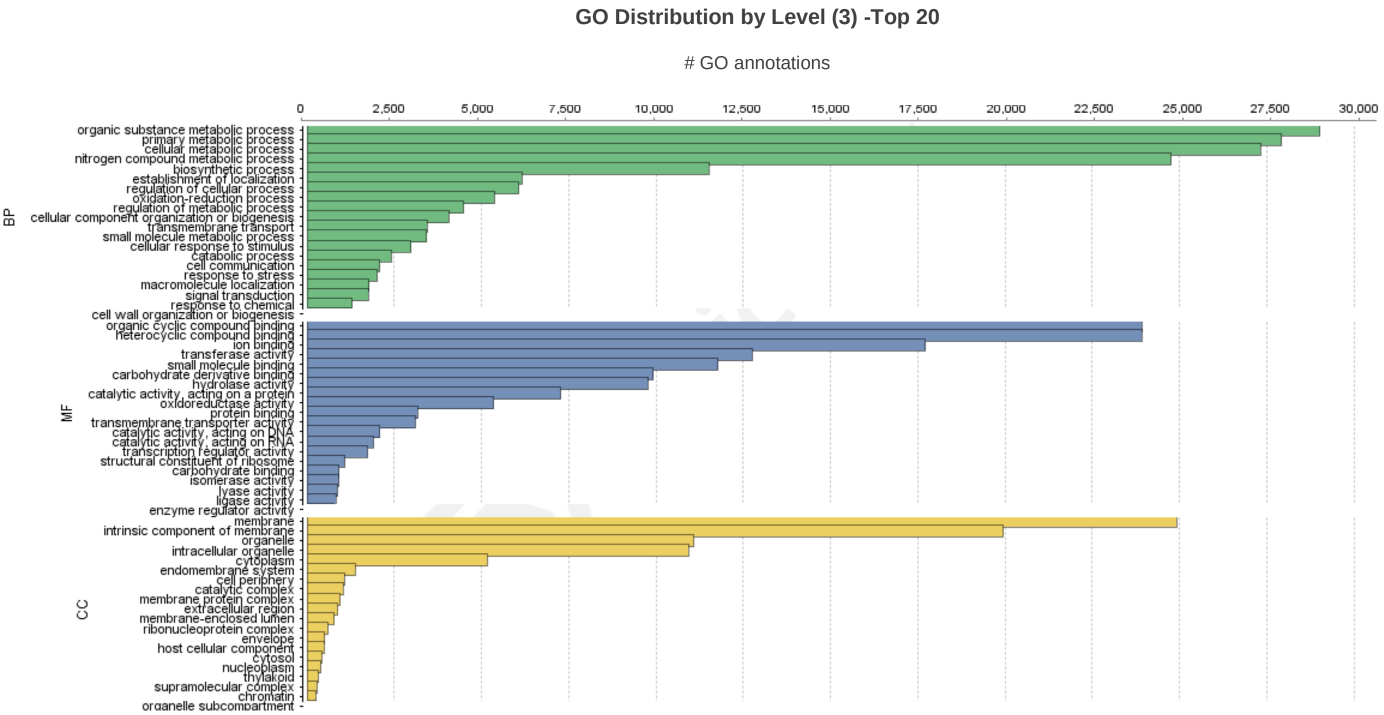

Supplement: Supplementary file 1 — Supplementary Figure S1. [file 41598_2021_83113_MOESM1_ESM.pdf]
